# Efficacy of nursing intervention using an adverse event predictive model for head and neck carbon-ion radiotherapy: A prospective clinical study

**DOI:** 10.1016/j.tipsro.2025.100364

**Published:** 2025-12-05

**Authors:** Chika Hirai, Atsushi Musha, Hirofumi Shimada, Yoko Kitada, Tatsuya Ohno

**Affiliations:** a Gunma University Heavy Ion Medical Center, 3-39-22, Showa-machi, Maebashi, Gunma 371-8511, Japan; b Gunma University Hospital Nursing, 3-39-22, Showa-machi, Maebashi, Gunma 371-8511, Japan; c Department of Oral and Maxillofacial Surgery and Plastic Surgery, Gunma University Graduate School of Medicine, 3-39-22, Showa-machi, Maebashi, Gunma 371-8511, Japan

**Keywords:** Radiation-induced toxicity, Nursing intervention, Carbon-ion radiotherapy, Quality of life, Head and neck tumor

## Abstract

**Introduction:**

Radiation dermatitis and oral mucositis are common acute toxicities from carbon-ion radiotherapy (CIRT) for head and neck cancers. These toxicities often impair quality of life (QOL) and can lead to treatment interruption. This study evaluated a dose surface model (DSM) shared by patients and nurses to determine whether it helped patients become more aware of their symptoms and improve their self-care.

**Methods:**

This prospective study enrolled 46 patients with head and neck malignancies who underwent CIRT between July 2017 and December 2019. The study program included nurse interviews and administration of the DSM-based patient self-care instructions, which were conducted before treatment, every week during CIRT, and at 1 and 2 months post-treatment. The self-care checklist and daily care frequency data were assessed. QOL was evaluated using the Short Form 8 at baseline, end of CIRT, and 2 months post-CIRT.

**Results:**

Radiation dermatitis occurred in 98% of the patients (grades 2–3 in 24%) and oral mucositis in 48% (grades 2–3). The self-care checklist scores improved significantly throughout the latter half of the treatment and post-treatment periods. Self-care frequency did not significantly correlate with adverse event severity, although mouth rinsing frequency tended to increase. Two months after treatment, QOL improved across several domains, particularly mental health.

**Conclusion:**

The DSM-based nursing intervention program effectively enhanced patient awareness and confidence in managing radiation-induced skin and mucosal toxicities. This strategy may enhance supportive care and QOL during CIRT for head and neck cancers.

## Highlights

• Dose surface model helped patients with carbon-ion radiotherapy adverse effects.
• Nursing intervention improved patient self-care awareness and quality of life.
• Dose surface model-based nursing intervention was an effective educational tool.

## Graphical abstract

**Figure:**
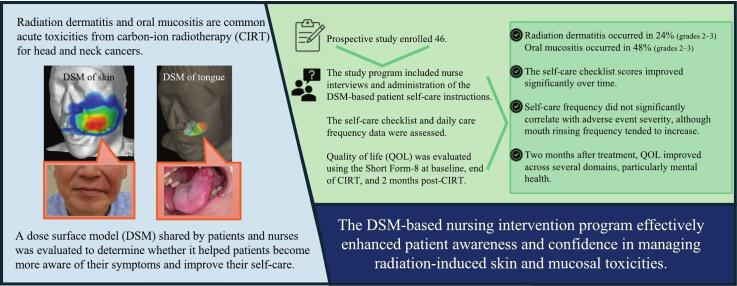

## Introduction

Head and neck cancers develop in a wide range of anatomical sites including oral and nasal cavities, paranasal sinuses, and the pharynx. Treatment-related adverse events can significantly affect speech, eating, breathing, and facial appearance, ultimately affecting patient quality of life (QOL). Although surgery remains the primary treatment for head and neck cancers, radiotherapy is increasingly considered as an organ-preserving option, dependent upon the extent of tumor invasion. However, even with radiotherapy, adverse events can unavoidably affect various critical organs.

Among radiotherapy modalities, carbon-ion radiotherapy (CIRT) is distinguished by its higher dose concentration and stronger cytotoxic effect than X-ray irradiation, leading to more localized adverse events. The reported acute adverse events for head and neck CIRT include grades 2–3 radiation dermatitis, which occurs in 31–44 % of patients, and grades 2–3 oral mucositis, which occurs in 47–66 % of patients, as classified by the Common Terminology Criteria for Adverse Events CTCAE version 4.0 [1], [2], [3].

Severe oral mucositis can severely impact QOL, causing increased pain and nutritional deficiencies and immune deterioration due to reduced food intake, which may ultimately lead to treatment interruption or discontinuation [4], [5], [6], [7], [8]. Daily care plays a crucial role in preventing severe cases of mucositis [9], [10], [11], [12], [13]. Musha et al. [14] introduced an approach to predict the onset of adverse events and integrated this information into daily care. Their analysis of 39 patients with head and neck cancers undergoing CIRT revealed an irradiation dose threshold capable of predicting both the location and severity of oral mucositis using a 3-dimensional (3D) model [14]. A similar technique, known as the dose surface model (DSM), has also been applied to model radiation-induced dermatitis [15]. Since patients often struggle to anticipate the location and severity of adverse events, this predictive approach may facilitate more effective management of treatment-related complications.

In this study, we implemented the DSM as a nursing intervention by sharing predictive information regarding the occurrence of oral mucositis and dermatitis with the patients. The effectiveness of the DSM on treatment, patient self-care behaviors, and quality of life and its clinical utility were evaluated.

## Materials and methods

### Patients

This study was approved by Gunma University Hospital Clinical Research Review Board and registered with the Ethics Review Committee for Medical Research Involving Human Subjects (approval date May 19, 2017; approval no. HS2016-120). All the procedures were conducted in accordance with the principles of the Declaration of Helsinki. Written informed consent was obtained from all the participants before CIRT initiation.

This prospective clinical study included 46 patients diagnosed with malignancies of the head and neck region who were treated with CIRT between July 2017 and December 2019 at our institution.

### Carbon-ion radiotherapy

The CIRT techniques and treatment planning have been previously described [1], [3]. The treatment regimen consisted of 16 fractions during approximately 4 weeks and was typically delivered 4 times per week.

### Dose surface model

**Fig. 1:**
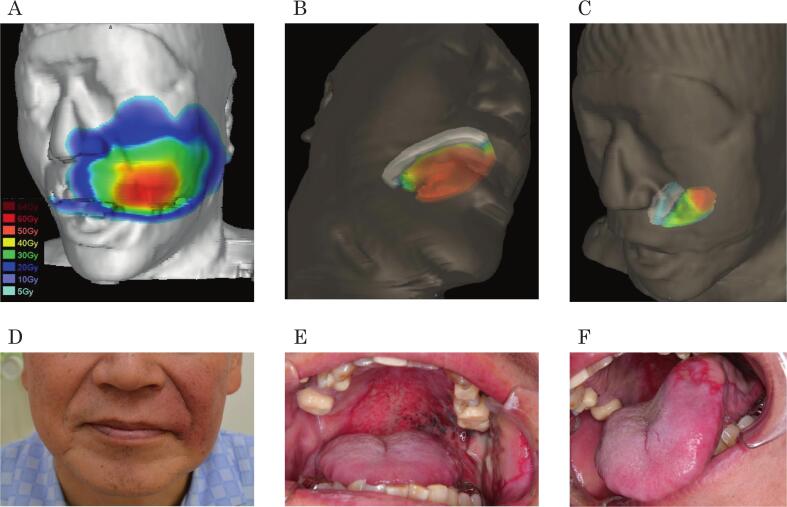
Dose surface model and corresponding adverse events. (A) Dose surface model of the skin, (B) dose surface model of the palate, (C) dose surface model of the tongue; red areas indicate higher dose regions and blue areas indicate lower dose regions. (D) Radiation dermatitis at 1 week after post-carbon-ion radiotherapy. (E) Palatal mucositis at 12 fractions. (F) Tongue mucositis at 12 fractions. (For interpretation of the references to colour in this figure legend, the reader is referred to the web version of this article.)

Specialized counseling using the DSM was provided to all patients prior to treatment, which focused on the risks of radiation-induced mucositis and dermatitis. The DSM approach was adapted from a previously published method [14]. Using MIM Maestro software (version 6.9.3, MIM Software Inc., Beachwood, OH, USA), 3D models of the oral mucosa (palate and tongue) and facial skin were created. Treatment plans from the XiO-N system (Elekta AB, Stockholm, Sweden) were exported to MIM for dose distribution visualization. High-dose areas were overlaid in color on 3D models to indicate the risk zones for mucositis and dermatitis. Fig. 1 illustrates the DSM in comparison to the observed adverse effects.

### Self-care check questionnaire

A self-administered self-care check questionnaire in Japanese (Supplementary Fig. S1) was developed to assess patient understanding of and engagement in skin and oral care. Five items were evaluated using a visual analog scale (VAS) at the following time points: before CIRT, every week during CIRT, and 1 and 2 months after treatment. The questions assessed patient understanding of (1) irradiated skin areas, (2) appropriate skin care, (3) possible mucositis sites, (4) oral care techniques, and (5) confidence in tooth brushing and gargling routines.

### Quality of life assessment

Health-related QOL was assessed using the Short Form 8 (SF-8) questionnaire [16], which includes 8 domains summarized from a Physical Component Score (PCS) and Mental Component Score (MCS). Higher scores indicate better QOL. The SF-8 was administered before CIRT, at the completion of 16 fractions, and 2 months post-treatment.

### Nursing intervention

The nurses had at least 5 years of experience. Their experience at the Heavy Ion Medical Center ranged from less than 1 year to approximately 8 years; care was standardized according to each CTCAE grade. Their roles during CIRT included treatment assistance, disease assessment, and care guidance. Before the study began, we conducted lectures to confirm their knowledge of the irradiation field in patients using the dose model and care for oral mucositis and dermatitis. These lectures were given by nurses well-versed in nursing education for radiation-related adverse events and covered fundamental aspects of oral care, skin care, and the dose model. This ensured standardized knowledge and methods for evaluating adverse events and care tailored to respond to adverse events based on existing literature [17], [18], [19]. Prior to CIRT initiation, physicians or nurses explained the risk areas for radiation-induced dermatitis and mucositis using the DSM visualizations (Fig. 1). The nurses provided instructions using a standardized booklet describing self-care techniques and precautions. The intervention team included a radiation oncologist for head and neck and nurses from the Heavy Ion Medical Center.

Nursing interviews were conducted at baseline, every week during CIRT, and 1 and 2 months after CIRT. Adverse events were graded using CTCAE version 4.0. Self-care frequency (including face washing, mouth rinsing, and tooth brushing) was recorded by the patients using self-report forms on treatment days and at 1 and 2 months post-CIRT. Nurses reviewed the DSM with patients, identified high-risk areas, and provided personalized feedback based on questionnaire responses.

**Fig. 2:**
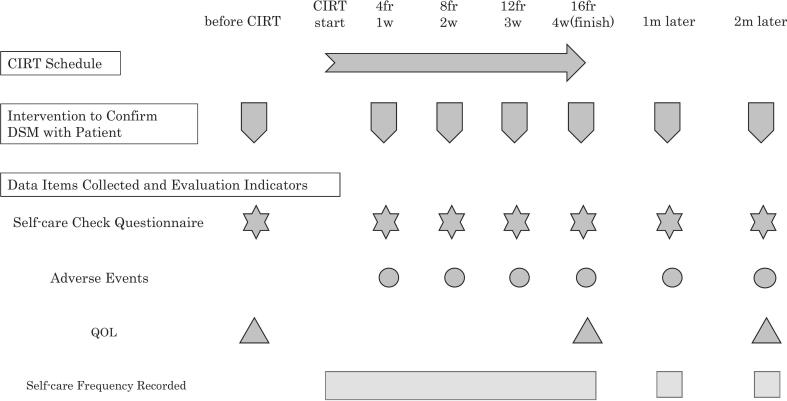
Nursing intervention and assessment schedule. The downward arrowheads represent DSM confirmation sessions. The right arrow indicates the CIRT treatment course. Stars represent self-care check questionnaires, circles denote adverse event evaluations, and triangles indicate QOL assessments. CIRT, carbon-ion radiotherapy; DSM, dose surface model; fr, fraction; m, months; QOL, quality of life; w, weeks.

Prior to the study initiation, a standardized training session was held for nurses to ensure uniform assessment and care protocols. The schedules for the nursing interventions, self-care assessments, and QOL surveys are displayed in Fig. 2.

### Statistical analyses

The data collected included responses to the self-care questionnaire, frequency and severity of radiation dermatitis and mucositis, patient characteristics, self-care frequency, and QOL scores.

The Friedman test was used to analyze changes in self-care scores across time points. Chi-square tests were used to examine the associations between self-care frequency and severity of mucositis and dermatitis. Significance was set at p < 0.05. All statistical analyses were conducted using SPSS Statistics software version 28 (IBM Corp., Armonk, NY, USA).

## Results

Table 1 lists the summary of patient and tumor characteristics. The patient median age was 67 years, and the man-to-woman ratio was equal. The most common primary tumor sites were the paranasal sinus (24 %), nasal cavity (22 %), and parotid gland (20 %). The most common histological types were adenoid cystic carcinoma (36 %) and malignant melanoma (21 %). Most patients were classified with T4 according to the Eighth Edition of the Union for International Cancer Control TNM staging system. For most patients, a total dose of 64.0 Gy (relative biological effectiveness [RBE]) was administered. Patients who required wide mucosal or skin irradiation received 57.6 Gy (RBE), whereas those with bone and soft tissue sarcomas received 70.4 Gy (RBE). One patient with bone and soft tissue sarcoma received a 67.2 Gy (RBE). Forty-one patients received primary treatment and 5 patients received recurrence treatment. Of those 5 patients, 3 had re-irradiation CIRT and 2 had recurrence postoperatively. Seven patients underwent cancer treatment for sites other than the head and neck (Table 1).

**Table 1:** Patient and tumor characteristics.

Total		N=46
Median age in years (range)		67.5 (25–85)
Sex n (%)	Men	20 (43)
	Women	26 (57)
Lesion site n (%)	Paranasal sinus	11 (24)
	Nasal cavity	10 (22)
	Parotid gland	9 (20)
	Oral cavity	7 (15)
	External auditory canal	3 (7)
	Others	6 (13)
Histologic type n (%)	Adenoid cystic carcinoma	17 (36)
	Malignant melanoma	10 (21)
	Bone and soft tissue tumors	5 (11)
	Adenocarcinoma	4 (9)
	Others	10 (22)
Total dose n (%)	70.4 Gy (RBE)	2 ((4)
	67.2 Gy (RBE)	1 (2)
	64 Gy (RBE)	33 (72)
	57.6 Gy (RBE)	10 (22)
T-stage n (%)	T0	1 (2)
	T1	5 (11)
	T2	3 (7)
	T3	10 (21)
	T4	27 (59)
N-stage n (%)	N0	43 (93)
	N1	1 (2)
	N2	2 (5)
M−stage n (%)	M0	45 (98)
	M1	1 (2)
Treatment n (%)	Primary treatment	41 (89)
	Recurrence treatment	5 (11)
Experience of other cancer treatment n (%)	Yes	7 (15)
	No	39 (85)

RBE, relative biological effectiveness.

**Fig. 3:**
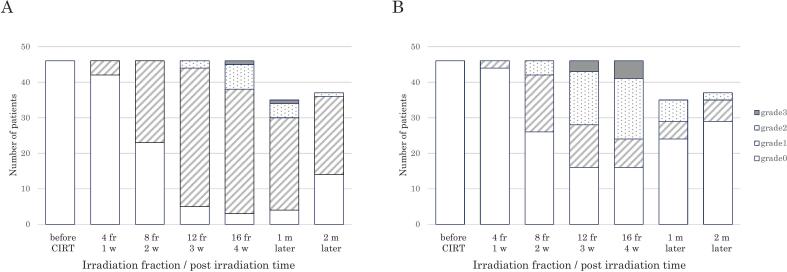
Incidences and severities of radiation dermatitis and oral mucositis. The y-axis represents the number of patients and the x-axis denotes the treatment timeline from before CIRT to 2 months post-treatment. (A) Radiation dermatitis. (B) Oral mucositis. White bars: grade 0; striped bars: grade 1; dotted bars: grade 2; black bars: grade 3. CIRT, carbon-ion radiotherapy; fr, fraction; m, months; w, weeks.

The frequencies and severities of radiation dermatitis and oral mucositis are summarized in Fig. 3. Radiation dermatitis was classified as grade 2 for 20 % and grade 3 for 4 % of the patients. Oral mucositis was grade 2 for 35 % and grade 3 for 13 % of the patients (Supplementary Table S1). Of the 6 patients with T0–1, grade 1 dermatitis was observed in 4 and grade 2 in 2, whereas 3 had a mucositis grade of 0, 2 had grade 1, and 1 had grade 2. Grade 3 events only occurred in the T3–4 patient group. N2 and N1 were very rare, in only 3 patients, with dermatitis grade 1 for 2 patients and grade 2 for 1 patient, and mucositis grade 0 for 1 patient and grade 2 for 2 patients. None of the patients had a grade 3 or higher for dermatitis or mucositis. Both adverse events began to appear after 4 fractions. Grade 1 radiation dermatitis was most frequently observed at 12 fractions, whereas grade 2 oral mucositis was most common at 16 fractions. All patients completed the entire CIRT course without treatment interruption.

The relationships between self-care frequency and severity of radiation dermatitis and oral mucositis are displayed in Supplementary Fig. S2. Face washing was typically performed 1–2 times daily throughout the treatment course. Mouthwash use averaged 4–6 times daily during CIRT and decreased to approximately 3 times daily immediately after treatment. Tooth brushing was performed approximately 3 times per day. Significant associations were not observed between face washing frequency and radiation dermatitis severity (p = 0.876), frequency of mouthwash use and oral mucositis severity (p = 0.136), or brushing frequency and mucositis severity (p = 0.617) (Supplementary Tables S2–S4).

**Fig. 4:**
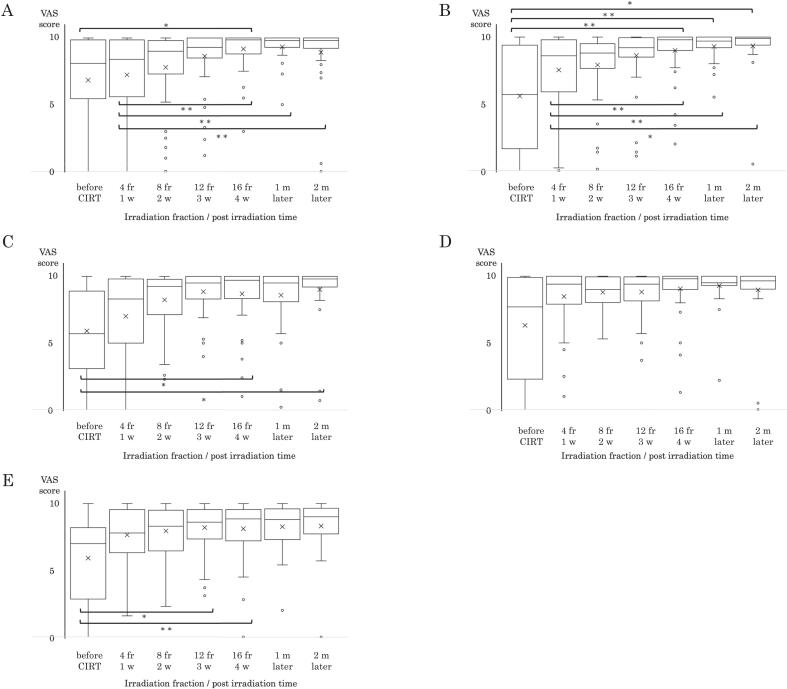
Longitudinal changes in self-care behavior. Self-care behavior was evaluated using the VAS scores for 5 questionnaire items over time. The y-axis indicates the VAS score, and the x-axis indicates the time course. (A) Understanding irradiated skin areas. (B) Understanding skin care methods. (C) Understanding likely mucositis sites. (D) Understanding oral care for irradiated areas. (E) Confidence in toothbrushing and gargling techniques. x: average scores. * p < 0.05, * * p < 0.01 CIRT, carbon-ion radiotherapy; fr, fraction; m, months; VAS, visual analog scale; w, weeks.

Fig. 4 illustrates the trends in self-care behaviors based on the self-care questionnaire (questions 1–5), measured using a VAS. Higher scores indicated stronger self-perceived understanding and performance.

Significant improvement in the irradiated skin areas was observed from baseline to 16 fractions (p < 0.05). The scores at 16 fractions and 1 and 2 months post-CIRT were significantly higher than those at 4 fractions (Fig. 4A). The scores for understanding skin care techniques increased significantly from baseline at 16 fractions and remained elevated 1 and 2 months post-treatment (Fig. 4B). The VAS scores for understanding mucositis-prone areas scores significantly increased at 16 fractions and 2 months post-CIRT compared to baseline (Fig. 4C). No significant changes were observed for understanding of oral care methods (Fig. 4D). Scores significantly increased for confidence in tooth brushing and gargling at 12 and 16 fractions compared to baseline (Fig. 4E).

Overall, greater variability in self-perceived understanding was observed before treatment, with stabilization during the latter half of CIRT. When comparing patients who received primary or recurrence treatment with the self-care questionnaire results, both patient groups generally had increasing VAS scores as treatment progressed (Supplementary Fig. S3). However, for the answers to question 1, perhaps due to prior irradiation history, there seemed to be a tendency for better understanding of the primary irradiation field by those with recurrence. Interestingly, after 2 months, the patients in the recurrence treatment group had higher VAS scores and improved understanding of all 5 questions compared to those in the primary treatment group. The patients who did and did not undergo cancer treatment for sites other than the head and neck generally had increasing VAS scores for self-care behavior as treatment progressed; there was no significant difference between these groups (Supplementary Fig. S3). The self-care behavior VAS results for men and women, did not significantly differ for any question or time point (Supplementary Fig. S4). Regarding VAS scores during treatment, the younger patients demonstrated significantly better understanding at several treatment time points for items related to predicted sites of dermatitis/oral mucositis onset and skin care methods (Supplementary Fig. S5).

**Fig. 5:**
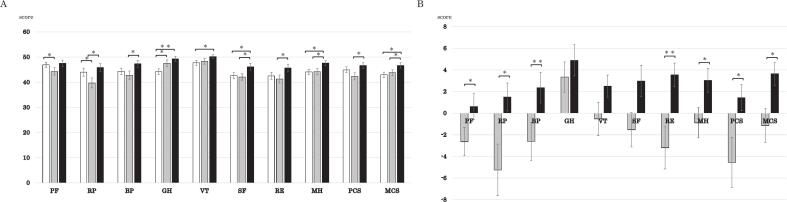
Temporal changes in SF-8 QOL scores. (A) Absolute SF-8 scores at each time point. (B) Changes in SF-8 scores relative to baseline. White bars: baseline; gray bars: 16 fractions; black bars: 2 months post-CIRT. BP, bodily pain; GH, general health; MCS, mental component score; MH, mental health; PCS, physical component score; PF, physical functioning; QOL, quality of life; RP, role physical; RE, role emotional; SF, social functioning; SF-8, Short Form 8; VT, vitality. * p < 0.05, * * p < 0.01.

Temporal changes in SF-8 domain scores are shown in Fig. 5A. All the domains remained below the national reference value of 50 throughout the observation period. Fig. 5B summarizes the score changes from baseline. Significant improvements were observed in the domains Physical Functioning, Role Physical, Bodily Pain, Role Emotional, Mental Health, PCS and MCS.

Physical Functioning significantly declined at the end of CIRT compared to baseline; the scores significantly recovered at 2 months post-treatment. Role Physical decreased at the end of CIRT and significantly improved at 2 months. Bodily Pain scores significantly improved from the end of CIRT to 2 months post-treatment. General Health significantly increased at the end of CIRT and at 2 months compared with baseline. Vitality gradually increased with significant improvement at 2 months post-CIRT compared with baseline. Social Functioning significantly improved from baseline to 2 months and from the end of CIRT to 2 months. Role Emotional declined at 16 fractions and significantly improved above baseline at 2 months. Mental Health significantly improved from baseline to 2 months and from the end of CIRT to 2 months. PCS declined at the end of CIRT, although improved beyond baseline levels at 2 months. MCS improved from the end of CIRT onward, with significant increases at 2 months compared with both baseline and at 16 fractions.

QOL outcomes related to treatment type (recurrence vs. initial treatment) were consistent with the trends observed for all patients (Supplementary Fig. S6). The only significant difference in the retreatment group were the Bodily Pain scores. There was no significant difference with patients who had undergone cancer treatment for sites other than the head and neck. Although the QOL scores had an upward trend after treatment for all patients, the items showing significant differences between women and men differed except for Role Emotional (Supplementary Fig. S7). For women, the items were Physical Functioning, Role Physical, Vitality, Role Emotional, Mental Health, and MCS. For men, Bodily Pain, Role Emotional, and PCS were dominant. Although there were no sex differences in the perception of the irradiation field during this intervention, the intervention had a stronger impact on mental QOL for women and a stronger impact on physical QOL for men. Notably, the same approach led to different perceptions and impacts on QOL based on sex. However, the positive effect on Role Emotional: Mental appeared consistent for both sexes. Regarding QOL, age comparisons revealed that for those aged < 67.5 years, QOL values increased significantly post-treatment, particularly for Role Emotional and MCS. Conversely, for those aged > 67.5 years, the Role Physical and PCS values increased (Supplementary Fig. S8).

## Discussion

Radiation dermatitis and oral mucositis, common adverse events of head and neck cancer radiotherapy, require daily care [11]. Implementing preventive care before initiating radiotherapy has been effective [10], [12]. Therefore, it is crucial for patients to consciously engage in self-care as part of their daily routine. In this study, we used the DSM as a nursing intervention program. Although the severity of dermatitis and oral mucositis did not significantly differ from previous studies that did not include this type of nursing intervention [1], [2], [3], a notable improvement was observed in patient awareness of self-care and an increase in their QOL scores. These findings suggest that a nursing intervention program using the DSM is beneficial.

In this study, dermatitis occurred in 98 % of the participants, with dermatitis grades 2–3 in 24 %, which is a rate comparable to or lower than the 31–44 % reported in previous studies [1], [2], [3]. Similarly, oral mucositis grades 2–3 were observed in 48 % of the participants, which is within the range of 47–66 % reported in previous studies [1], [2], [3]. However, direct comparisons with previous studies are challenging because of variations in the irradiated areas, such as cases involving the external auditory canal that did not include the oral cavity.

Significant changes were not observed in face washing frequency, indicating that patients were able to maintain their usual self-care routines. Although the number of mouth rinses did not show a significant correlation with oral mucositis severity, there was a trend toward increased mouthwash use during the treatment period. Kartin et al. [11] reported that patients who received regular oral care had a reduced incidence of oral mucositis. Additionally, Musha et al. [9] suggested that mucositis may not only be influenced by the radiation dose but also by the presence of oral bacteria, emphasizing the importance of oral care in preventing severe mucositis. Based on these findings, a nursing intervention program using the DSM may help maintain appropriate frequency of self-care, potentially enabling all patients to complete CIRT without experiencing severe dermatitis or oral mucositis. Interventions before the onset of adverse events may lead to early detection and therapeutic intervention, enabling CIRT to be completed without serious illness.

The results of this study suggest that self-care behavior interventions were effective in increasing patient awareness of self-care and providing psychological support compared to before CIRT initiation. By repeatedly using the DSM, nurses confirmed the irradiation area, enabling patients to gradually recognize it themselves. Although it is possible that the patients identified the irradiation area due to the onset of dermatitis and oral mucositis, DSM use before CIRT may have helped them visually identify these areas in advance. Additionally, the patients learned about skin and oral self-care throughout the CIRT period. Continuous nursing interventions allowed patients to understand the irradiated area and develop self-care skills for their skin and oral cavity during CIRT, leading to greater self-care awareness. Alterio et al. [10] reported that the incidence and duration of oral mucositis decreased when patients received accurate oral hygiene assessments before radiotherapy, frequent evaluations, and clear symptom relief protocols [10]. The Mucositis Study Group of the MASCC/ISOO also emphasized the importance of “patient education” serving as a new guideline [20]. In this study, we used the DSM to explain the risk areas for oral mucositis to each patient and continuously provided guidance on oral mucosa care. This strategy aligns with the patient education recommendations in the Mucositis Study Group guidelines, suggesting that it may be an effective approach for basic oral care. Miaskowski et al. [21] noted that nurses who provided psychological support while utilizing patients’ learning abilities to reduce cancer pain, led to improved pain management and symptom relief. A series of nursing interventions, including repeated identification and assessment of dermatitis and oral mucositis onset areas using the DSM before treatment and continuous feedback on self-care, helped to monitor patient conditions and provided psychological support, which proved beneficial.

Patient background may influence VAS score variability. Although factors such as sex or prior cancer treatment outside the head and neck region appeared to have little impact, recurrence treatment for the same site tended to allow easier understanding of the treatment area early on compared to the understanding by patients undergoing initial treatment. Moreover, the younger patients tended to demonstrate understanding earlier. The importance of timing nursing interventions and understanding patient backgrounds was confirmed.

Most QOL scores decline after both CIRT and surgery [3], [22], [23]; however, the nursing intervention in this study contributed to faster recovery of QOL scores. Compared to pre-CIRT levels, the QOL scores improved 2 months post-CIRT. In contrast, QOL scores after head and neck cancer surgery generally improve within 3–6 months post-treatment [22], [23], suggesting that CIRT may enable earlier QOL recovery than surgery. Compared with prospective clinical trials of CIRT for patients with non-squamous cell carcinoma [3], this study showed a trend toward faster QOL recovery, particularly for MCS, from pre-CIRT to 2 months post-CIRT. Although the severity of the adverse events remained similar, these findings indicate that nursing intervention played a crucial role in supporting patient mental health. Zhang et al. [5] stated that team-based medical care promotes treatment adherence, optimizes care plans, and enhances posttreatment QOL and survival. Additionally, Chang et al. [24] noted that when patients fully understand their treatment plan alongside their healthcare providers, their mental and physical well-being improves; therefore, they emphasize the importance of providing sufficient information [24].

Notably, the results of this study also suggest patient background may influence QOL differently. Although recurrence treatment and treatment history for other cancers appeared to have little impact on QOL, the QOL indicators more susceptible to influence based on sex and age suggest that treatment and nursing needs vary depending on patient backgrounds. This highlights the importance for medical staff, especially cancer treatment related staff, to understand and accommodate these diversities. The DSM can serve as a communication tool that fosters a shared understanding of potential adverse events between patients and healthcare providers, positively affecting patient mental health. Furthermore, the DSM allowed nurses to implement standardized interventions, improve the quality of nursing guidance, and enhance their knowledge, which likely contributed to patient reassurance.

This study has several limitations. First, the observed differences in self-care behavior changes coinciding with adverse event onset suggest that a significant difference would have occurred even without DSM use. However, because the intervention was implemented before adverse events appeared, we only considered the effects of the DSM-based nursing intervention. Second, the teaching methods using mucosal models, adverse event assessment, and care instruction for the nurses were conducted according to standardized procedures; however, biases may have arisen based on factors such as the nurses' years of clinical experience. Third, the self-care check questionnaire has not been validated. Active validation through multicenter prospective research studies is necessary and subsequent changes to the questionnaire content may be required. Finally, this study focused solely on acute adverse events; therefore, future clinical studies should examine late-onset adverse events.

## Conclusion

A series of nursing interventions, including periodic DSM-based identification of the onset site, dermatitis and oral mucositis assessments, and daily care feedback, did not reduce dermatitis and oral mucositis incidences. However, these interventions significantly increased patient awareness of self-care behaviors and had a positive impact on their mental well-being, as reflected by their improved QOL scores. This effect can be attributed to regular nursing interviews, standardized nursing care, and a multidisciplinary team approach. Nursing interventions using the DSM were effective in enhancing patient self-care awareness and providing them with a sense of reassurance and comfort throughout their treatment. The findings of this study are expected to improve the quality of radiotherapy for head and neck cancers, potentially leading to a more patient-centered care.

## CRediT authorship contribution statement

**Chika Hirai:** Conceptualization, Data curation, Formal analysis, Investigation, Methodology, Project administration, Resources, Visualization, Writing – original draft, Writing – review & editing. **Atsushi Musha:** Conceptualization, Data curation, Formal analysis, Funding acquisition, Investigation, Methodology, Project administration, Resources, Software, Supervision, Validation, Visualization, Writing – original draft, Writing – review & editing. **Hirofumi Shimada:** Methodology, Resources, Software, Writing – review & editing. **Yoko Kitada:** Investigation, Writing – review & editing. **Tatsuya Ohno:** Supervision, Writing – review & editing.

## Funding

This work was supported by JSPS KAKENHI (grant number: 25 K10880) and the Takeda Science Foundation. The study sponsors were not involved in the study design; data collection, analysis, or interpretation; manuscript writing; or decision to submit the manuscript for publication.

## Declaration of competing interest

The authors declare that they have no known competing financial interests or personal relationships that could have appeared to influence the work reported in this paper.

## Data Availability

The data that supports the findings of this study are available within the article.

## References

[1] Musha A., Kubo N., Okano N., Kaminuma T., Kawamura H., Sato H. (2022). Prospective observational study of carbon-ion radiotherapy for non-squamous cell carcinoma of the head and neck in Gunma University. J Oral Maxillofac Surg Med Pathol.

[2] Mizoe J.E., Hasegawa A., Jingu K., Takagi R., Bessyo H., Morikawa T. (2012). Results of carbon ion radiotherapy for head and neck cancer. Radiother Oncol.

[3] Shirai K., Saitoh J.I., Musha A., Abe T., Kobayashi D., Takahashi T. (2017). Prospective observational study of carbon-ion radiotherapy for non-squamous cell carcinoma of the head and neck. Cancer Sci.

[4] Ogama N., Suzuki S. (2012). Adverse effects and appetite suppression associated with particle beam therapy in patients with head and neck cancer. Jpn J Nurs Sci.

[5] Zhang Z., Tian L., Liu J., Jiang H., Wang P. (2024). Evidence summary on managing radiotherapy-induced oral mucositis in patients with head and neck cancer. Asia Pac J Oncol Nurs.

[6] Ogama N., Ogama N. (2013). Development of an oral assessment tool to evaluate appetite in patients with head and neck cancer receiving radiotherapy. Eur J Oncol Nurs.

[7] Bansal M., Mohanti B.K., Shah N., Chaudhry R., Bahadur S., Shukla N.K. (2004). Radiation related morbidities and their impact on quality of life in head and neck cancer patients receiving radical radiotherapy. Qual Life Res.

[8] Ruo Redda M.G., Allis S. (2006). Radiotherapy-induced taste impairment. Cancer Treat Rev.

[9] Musha A., Hirai C., Kitada Y., Tsunoda A., Shimada H., Kubo N. (2022). Relationship between oral mucositis and the oral bacterial count in patients with head and neck cancer undergoing carbon ion radiotherapy: a prospective study. Radiother Oncol.

[10] Alterio D., Jereczek-Fossa B.A., Fiore M.R., Piperno G., Ansarin M., Orecchia R. (2007). Cancer treatment-induced oral mucositis. Anticancer Res.

[11] Kartin P.T., Tasci S., Soyuer S., Elmali F. (2014). Effect of an oral mucositis protocol on quality of life of patients with head and neck cancer treated with radiation therapy. Clin J Oncol Nurs.

[12] Ogama N., Suzuki S., Umeshita K., Kobayashi T., Kaneko S., Kato S. (2010). Appetite and adverse effects associated with radiation therapy in patients with head and neck cancer. Eur J Oncol Nurs.

[13] Westbury C., Hines F., Hawkes E., Ashley S., Brada M. (2000). Advice on hair and scalp care during cranial radiotherapy: a prospective randomized trial. Radiother Oncol.

[14] Musha A., Shimada H., Shirai K., Saitoh J.I., Yokoo S., Chikamatsu K. (2015). Prediction of acute radiation mucositis using an oral mucosal dose surface model in carbon ion radiotherapy for head and neck tumors. PLoS One.

[15] Kubo N., Kubota Y., Oike T., Kawamura H., Sakai M., Imamura A. (2020). Skin dose reduction by layer-stacking irradiation in carbon ion radiotherapy for parotid tumors. Front Oncol.

[16] Fukuhara S, Suzukamo Y. Manual of the SF-8. Japanese version. Kyoto: Qualitest Inc.; 2004.

[17] Harris D.J., Eilers J., Harriman A., Cashavelly B.J., Maxwell C. (2008). Putting evidence into practice: evidence-based interventions for the management of oral mucositis. Clin J Oncol Nurs.

[18] McQuestion M. (2011). Evidence-based skin care management in radiation therapy: clinical update. Semin Oncol Nurs.

[19] Marquez C.M., Wong W. (2021). ASTRO editorial: ONS guidelines for cancer treatment-related radiodermatitis. Pract Radiat Oncol.

[20] Elad S., Cheng K.K.F., Lalla R.V., Yarom N., Hong C., Logan R.M. (2020). MASCC/ISOO clinical practice guidelines for the management of mucositis secondary to cancer therapy. Cancer.

[21] Miaskowski C., Dodd M., West C., Schumacher K., Paul S.M., Tripathy D. (2004). Randomized clinical trial of the effectiveness of a self-care intervention to improve cancer pain management. J Clin Oncol.

[22] Miyoshi M., Fukuhara T., Kataoka H., Hagino H. (2016). Relationship between quality of life instruments and phonatory function in tracheoesophageal speech with voice prosthesis. Int J Clin Oncol.

[23] Suzuki K., Nishio N., Kimura H., Tokura T., Kishi S., Ozaki N. (2023). Comparison of quality of life and psychological distress in patients with tongue cancer undergoing a total/subtotal glossectomy or extended hemiglossectomy and free flap transfer: a prospective evaluation. Int J Oral Maxillofac Surg.

[24] Chang Y.L., Lee S.C., Liao C.T., Wang C.H., Lin Y.F., Chen S.C. (2020). Factors impacting on discordance with treatment plan in head and neck cancer patients: a retrospective, population-based cohort study. Support Care Cancer.

