# Supplementary material for: Efficacy of nursing intervention using an adverse event predictive model for head and neck carbon-ion radiotherapy: A prospective clinical study
Source: Tech Innov Patient Support Radiat Oncol. 2025 Dec 5;37:100364. doi: 10.1016/j.tipsro.2025.100364 (PMC12754237; doi:10.1016/j.tipsro.2025.100364)
Supplement: Supplementary Data 1 [file mmc1.pdf]

セルフケアチェック用紙 Self-Care Check Questionnaire

（　　　　　）回目 times・ヶ月後 month later

番号（　　　　　　　　）

〈↓印記入例〉Example of ↓mark entry

あなたの現状を 0～10 で評価し線を引いてください。Please rate your current situation on a scale of 0 to 10 and draw a line.

0 から 10 までの尺度の中でご自身の回答にあてはまる位置に↓印を記入してください。Please place a ↓ mark where it applies to your answer on a scale from 0 to 10.

0____________________↓____________________10

全く分からない I have no idea.　　　　　　　よく分かる I know exactly what I'm talking about.

1.　皮膚に照射している（照射する・していた）場所が分かる。

The place where the skin is irradiated (or was irradiated) can be identified.

0____10

全く分からない I have no idea.　　　　　　　よく分かる I know exactly what I'm talking about.

2.　照射している（照射する・していた）皮膚のケアの仕方が分かる。

You know how to take care of the skin that is (is/was) irradiated.

0____10

全く分からない I have no idea.　　　　　　　よく分かる I know exactly what I'm talking about.

3.　口の中の粘膜炎がおきる可能性がある場所が分かる。

Identify areas in the mouth where mucositis may occur.

0____10

全く分からない I have no idea.　　　　　　　よく分かる I know exactly what I'm talking about.

4.　照射している（照射する・していた）口の中のケアの仕方が分かる。

You know how to take care of the mouth that is (is/was) irradiated.

0____10

全く分からない I have no idea.　　　　　　　よく分かる I know exactly what I'm talking about.

5.　歯磨きやうがいの仕方に自信がある。

Confident in brushing and gargling.

0____10

全く自信がない I am not sure at all.　　　　　　　とても自信がある Very confident.
