# Supplementary material for: Efficacy of nursing intervention using an adverse event predictive model for head and neck carbon-ion radiotherapy: A prospective clinical study
Source: Tech Innov Patient Support Radiat Oncol. 2025 Dec 5;37:100364. doi: 10.1016/j.tipsro.2025.100364 (PMC12754237; doi:10.1016/j.tipsro.2025.100364)
Supplement: Supplementary Data 2 [file mmc2.pdf]

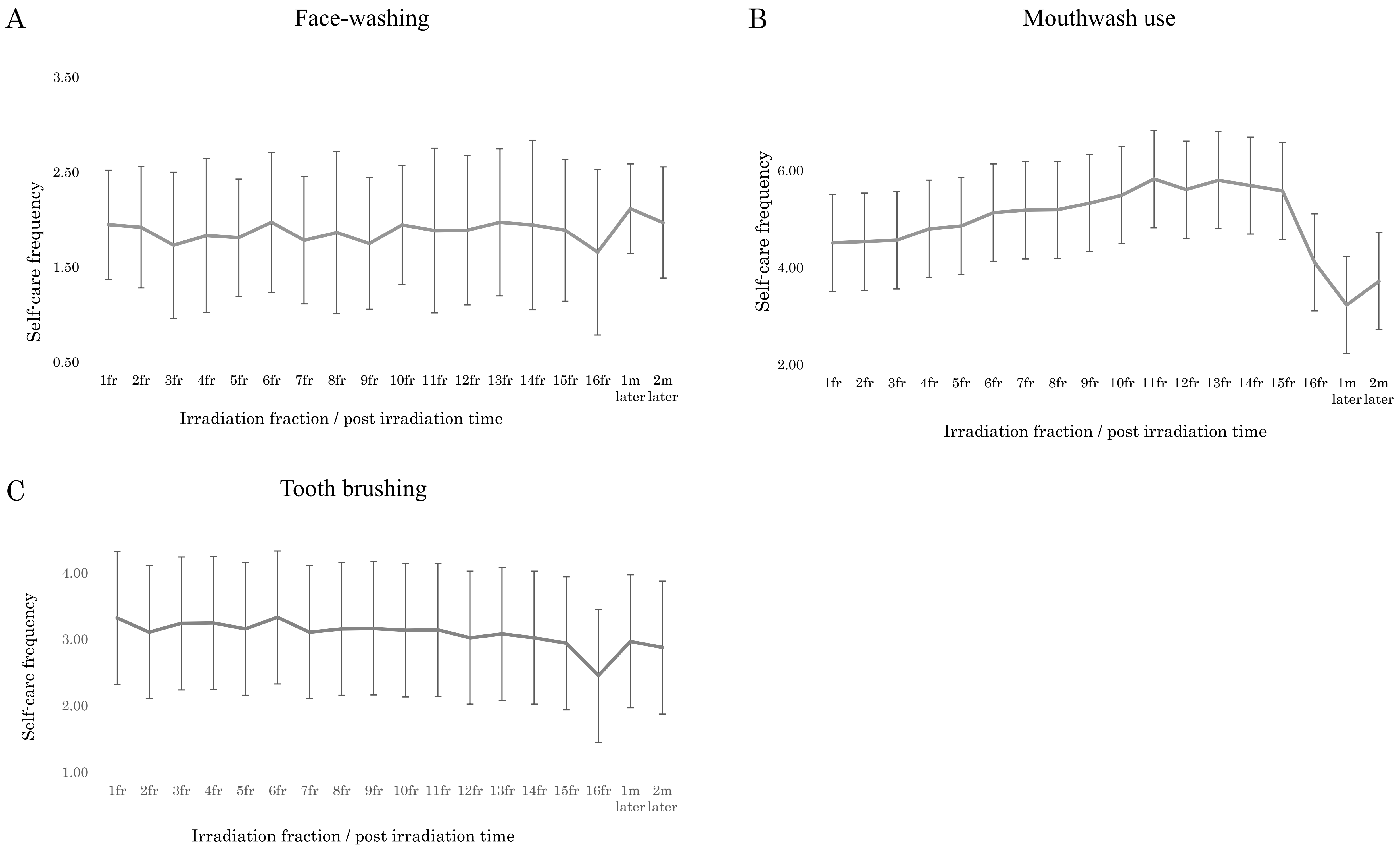

Supplementary Figure S2. Frequency of self-care behaviors

(A) Face-washing. (B) Mouthwash use. (C) Tooth brushing. The y-axis indicates the frequency of the self-care activities, and the x-axis denotes the timeline from the first fraction to 2 months post-CIRT. CIRT, carbon-ion radiotherapy; fr, fraction; m, months.
