# Supplementary material for: Efficacy of nursing intervention using an adverse event predictive model for head and neck carbon-ion radiotherapy: A prospective clinical study
Source: Tech Innov Patient Support Radiat Oncol. 2025 Dec 5;37:100364. doi: 10.1016/j.tipsro.2025.100364 (PMC12754237; doi:10.1016/j.tipsro.2025.100364)
Supplement: Supplementary Data 5 [file mmc5.pdf]

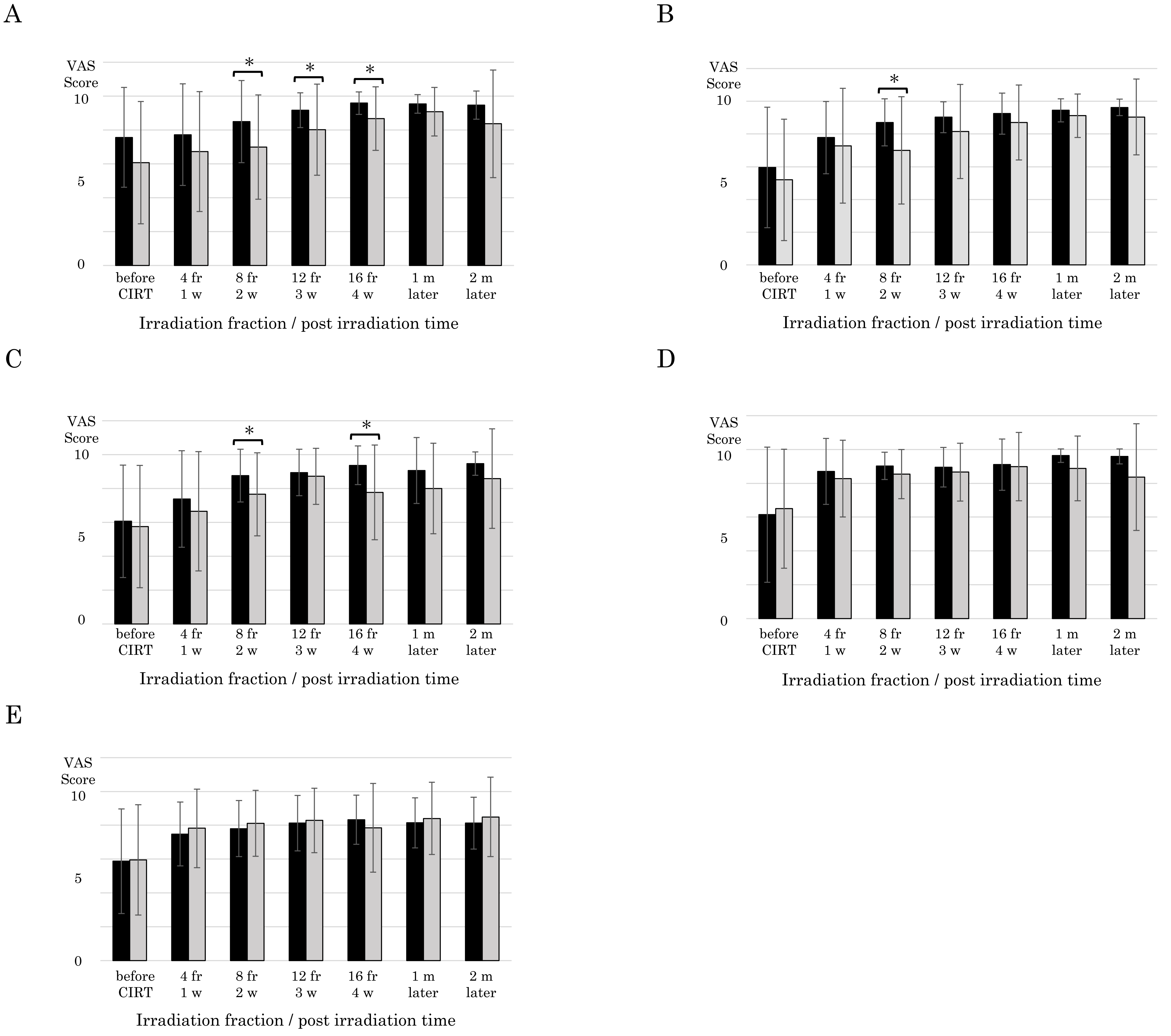

Supplementary Figure S5. Comparisons of changes in self-care behavior over time for age.

Self-care behavior was evaluated using the VAS scores for 5 questionnaire items over time. The y-axis indicates the VAS score, and the x-axis indicates the time course. (A) Understanding irradiated skin areas. (B) Understanding skin care methods. (C) Understanding likely mucositis sites. (D) Understanding oral care for irradiated areas. (E) Confidence in toothbrushing and gargling techniques. x: average scores. * $p < 0.05$

Black bars: patients aged <67.5 years; gray bars: patients aged >67.5 years. CIRT, carbon-ion radiotherapy; fr, fraction; m, months; VAS, visual analog scale; w, weeks.
