# Supplementary material for: Efficacy of nursing intervention using an adverse event predictive model for head and neck carbon-ion radiotherapy: A prospective clinical study
Source: Tech Innov Patient Support Radiat Oncol. 2025 Dec 5;37:100364. doi: 10.1016/j.tipsro.2025.100364 (PMC12754237; doi:10.1016/j.tipsro.2025.100364)
Supplement: Supplementary Data 8 [file mmc8.pdf]

A

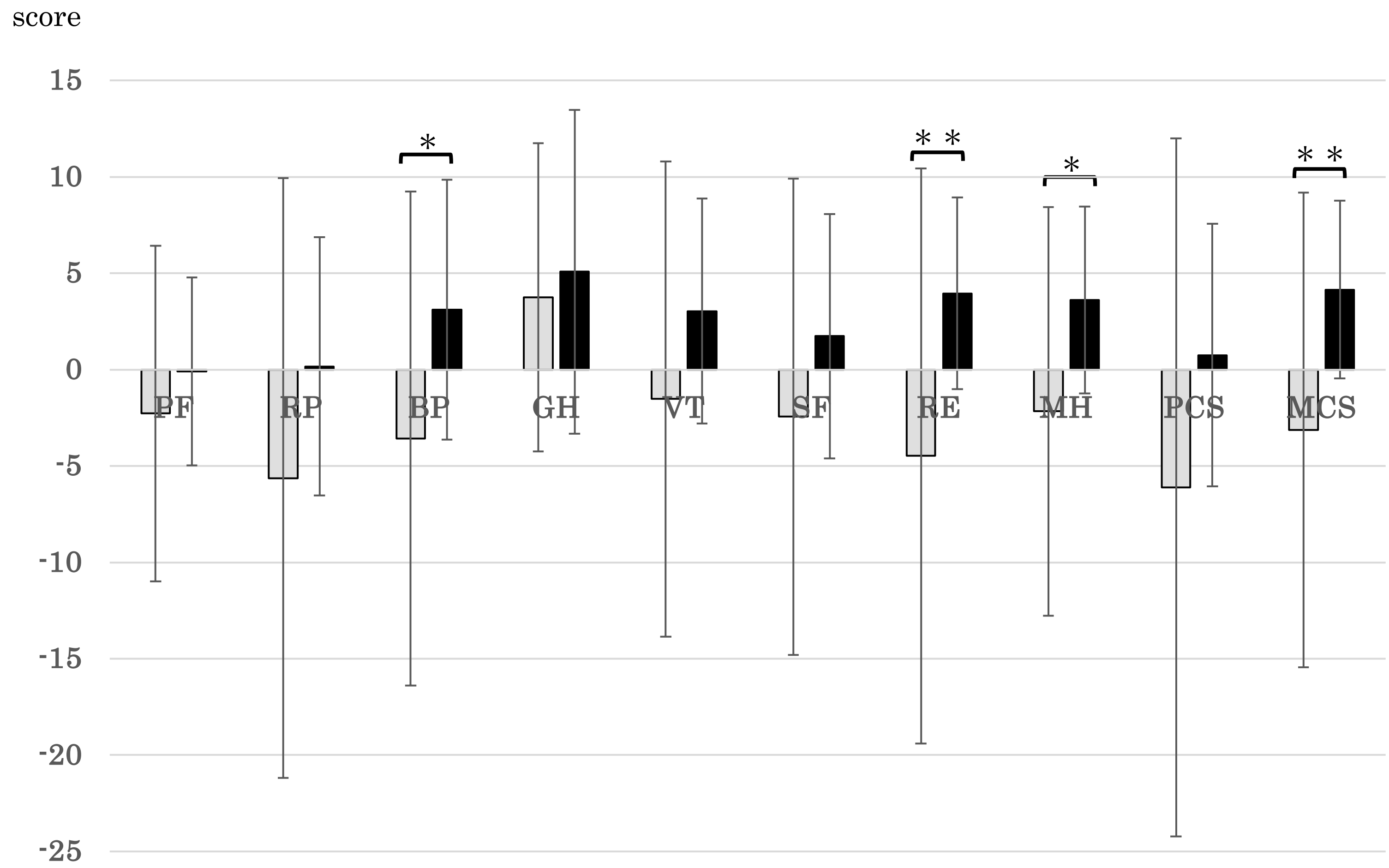

B

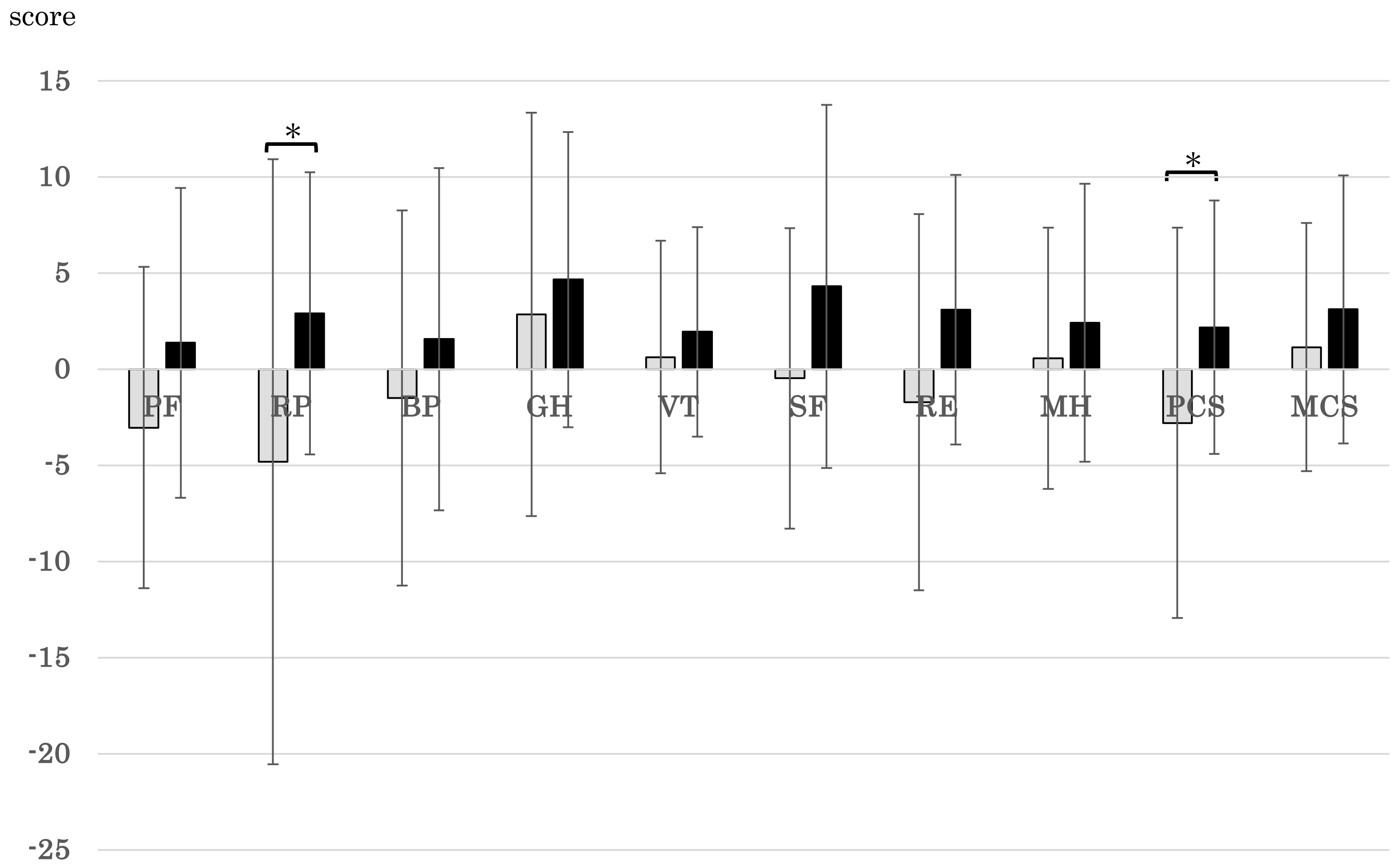

Supplementary Figure S8. Changes in SF-8 scores relative to baseline for age.

(A) patients aged <67.5 years. (B) patients aged >67.5 years.
Gray bars: 16 fractions; black bars: 2 months post-CIRT. BP, bodily pain; GH, general health; MCS, mental component score; MH, mental health; PCS, physical component score; PF, physical functioning; QOL, quality of life; RE, role emotional; RP, role physical; SF, social functioning; SF-8, Short Form 8; VT, vitality. * $p < 0.05$, * * $p < 0.01$.
