# Supplementary material for: Efficacy of nursing intervention using an adverse event predictive model for head and neck carbon-ion radiotherapy: A prospective clinical study
Source: Tech Innov Patient Support Radiat Oncol. 2025 Dec 5;37:100364. doi: 10.1016/j.tipsro.2025.100364 (PMC12754237; doi:10.1016/j.tipsro.2025.100364)
Supplement: Supplementary Data 9 [file mmc9.docx]

**Supplementary Table S1.** Summary of adverse events.

| n (%) | Grade 0 | Grade 1 | Grade 2 | Grade 3 |
| --- | --- | --- | --- | --- |
| Radiation dermatitis | 1 (2) | 34 (74) | 9 (20) | 2 (4) |
| Oral mucositis | 16 (35) | 8 (17) | 16 (35) | 6 (13) |

The p values: face washing (p = 0.876), mouthwash (p = 0.136), and tooth brushing (p = 0.617).
