# Supplementary material for: Efficacy of nursing intervention using an adverse event predictive model for head and neck carbon-ion radiotherapy: A prospective clinical study
Source: Tech Innov Patient Support Radiat Oncol. 2025 Dec 5;37:100364. doi: 10.1016/j.tipsro.2025.100364 (PMC12754237; doi:10.1016/j.tipsro.2025.100364)
Supplement: Supplementary Data 11 [file mmc11.docx]

**Supplementary Table S3.** Maximum adverse event grades and average mouthwash frequency.

|  | Self-care frequency average value | | | | Total |
| --- | --- | --- | --- | --- | --- |
|  | 0－2 | 3–4 | Over 5 | Not subject |  |
| Grades 0–1 | 3 | 8 | 13 | 0 | 24 |
| Grades 2–3 | 0 | 10 | 10 | 2 | 22 |
| Total | 3 | 18 | 23 | 2 | 46 |
